# Supplementary material for: CRISPR-based functional genomics in human dendritic cells
Source: eLife. 2021 Apr 27;10:e65856. doi: 10.7554/eLife.65856 (PMC8104964; doi:10.7554/eLife.65856)
Supplement: Transparent reporting form [file elife-65856-transrepform.docx]

***eLife’s* transparent reporting form**

We encourage authors to provide detailed information *within their submission* to facilitate the interpretation and replication of experiments. Authors can upload supporting documentation to indicate the use of appropriate reporting guidelines for health-related research (see [EQUATOR Network](http://www.equator-network.org/%20)), life science research (see the [BioSharing Information Resource](https://biosharing.org/)), or the [ARRIVE guidelines](http://www.plosbiology.org/article/info:doi/10.1371/journal.pbio.1000412) for reporting work involving animal research. Where applicable, authors should refer to any relevant reporting standards documents in this form.

If you have any questions, please consult our Journal Policies and/or contact us: [editorial@elifesciences.org](mailto:editorial@elifesciences.org).

**Sample-size estimation**

- You should state whether an appropriate sample size was computed when the study was being designed
- You should state the statistical method of sample size computation and any required assumptions
- If no explicit power analysis was used, you should describe how you decided what sample (replicate) size (number) to use

Please outline where this information can be found within the submission (e.g., sections or figure legends), or explain why this information doesn’t apply to your submission:

No sample-size calculation was performed in advance. All results were reproduced in cells from multiple independent donors, following conventions of the field. Within independent experiments, assays were performed in duplicate or triplicate following conventions of the field. This information is included in the methods section and the figure legends.

**Replicates**

- You should report how often each experiment was performed
- You should include a definition of biological versus technical replication
- The data obtained should be provided and sufficient information should be provided to indicate the number of independent biological and/or technical replicates
- If you encountered any outliers, you should describe how these were handled
- Criteria for exclusion/inclusion of data should be clearly stated
- High-throughput sequence data should be uploaded before submission, with a private link for reviewers provided (these are available from both GEO and ArrayExpress)

Please outline where this information can be found within the submission (e.g., sections or figure legends), or explain why this information doesn’t apply to your submission:

All main findings were derived from experiments with cells from at least 2 independent donors. The main hits from the genetic screen were validated in cells from 2 additional, independent donors. All treatments were performed in duplicate for readout by ELISA and qPCR and in duplicate or triplicate for readout by RNA-seq. Cell counts were generally conducted in duplicate. Information on replicates is contained in the figure legends.

For identification of differentially expressed genes in RNA-seq, only genes with an average count >2 across all conditions were included for analysis. During analysis of editing outcomes, some amplicons contained large deletions with boundaries >20 bp from an sgRNA cut site that were classified as “large deletions” by knock-knock but likely instead reflect amplification of partially complementary fragments, given in particular the rare occurrence of large deletions with individual sgRNAs. To avoid overestimating editing efficiency, reads with at least one alignment boundary >40 bp from an sgRNA cut site or both alignment boundaries >20 bp from sgRNA cut sites were re-classified into the “malformed layout” category. For analysis of editing outcomes for the genetic screen, only samples with >100 size-corrected on-target reads were included. This information is described in the methods.

**Statistical reporting**

- Statistical analysis methods should be described and justified
- Raw data should be presented in figures whenever informative to do so (typically when N per group is less than 10)
- For each experiment, you should identify the statistical tests used, exact values of N, definitions of center, methods of multiple test correction, and dispersion and precision measures (e.g., mean, median, SD, SEM, confidence intervals; and, for the major substantive results, a measure of effect size (e.g., Pearson's r, Cohen's d)
- Report exact p-values wherever possible alongside the summary statistics and 95% confidence intervals. These should be reported for all key questions and not only when the p-value is less than 0.05.

Please outline where this information can be found within the submission (e.g., sections or figure legends), or explain why this information doesn’t apply to your submission:

This information is described in the corresponding figure legends.

(For large datasets, or papers with a very large number of statistical tests, you may upload a single table file with tests, Ns, etc., with reference to sections in the manuscript.)

**Group allocation**

- Indicate how samples were allocated into experimental groups (in the case of clinical studies, please specify allocation to treatment method); if randomization was used, please also state if restricted randomization was applied
- Indicate if masking was used during group allocation, data collection and/or data analysis

Please outline where this information can be found within the submission (e.g., sections or figure legends), or explain why this information doesn’t apply to your submission:

Group allocation does not apply to this submission because there were no blinded experiments. No randomization was used.

**Additional data files (“source data”)**

- We encourage you to upload relevant additional data files, such as numerical data that are represented as a graph in a figure, or as a summary table
- Where provided, these should be in the most useful format, and they can be uploaded as “Source data” files linked to a main figure or table
- Include model definition files including the full list of parameters used
- Include code used for data analysis (e.g., R, MatLab)
- Avoid stating that data files are “available upon request”

Please indicate the figures or tables for which source data files have been provided:

Editing outcomes for all experiments and ELISA data for the genetic screen are provided in the form of supplemental files because they are referred to across multiple figures.
